# The Long-Term Costs of Family Trajectories: Women’s Later-Life Employment and Earnings Across Europe

**DOI:** 10.1007/s13524-020-00874-8

**Published:** 2020-04-23

**Authors:** Joanne S. Muller, Nicole Hiekel, Aart C. Liefbroer

**Affiliations:** 1 Netherlands Interdisciplinary Demographic Institute (NIDI), PO Box 11650, 2502 AR The Hague, The Netherlands; 2 University of Groningen, PO Box 72, 9700 AB Groningen, The Netherlands; 3 Department of Sociology and Social Psychology, University of Cologne, Albertus-Magnus-Platz, 50923 Cologne, Germany; 4 Department of Epidemiology, University Medical Center Groningen (UMCG), University of Groningen, PO Box 30001, 9700 RB Groningen, The Netherlands; 5 Department of Sociology, Vrije Universiteit Amsterdam, De Boelelaan 1081, 1081 HV Amsterdam, The Netherlands

**Keywords:** Life course, Later-life earnings, Later-life employment, Family trajectory, Comparative motherhood penalty

## Abstract

The “motherhood earnings penalty” is a well-established finding in many Western countries. However, a divide between mothers and nonmothers might oversimplify reality given that the family life course has diversified over the last decades. In addition, whether family choices have consequences for women’s employment and earnings in later life is not well known, particularly in a comparative perspective. Using data on 50- to 59-year-old women from the Generations and Gender Programme, the British Household Panel Survey, and SHARELIFE for 22 European countries, we derive a typology of women’s family trajectories and estimate its association with women’s later-life employment and earnings. Whereas family trajectory–related differences with regard to employment were relatively small, our findings reveal a clear, long-lasting family trajectory gradient in earnings. Childless women (with or without a partner) as well as single mothers had higher personal earnings than women whose family trajectories combined parenthood and partnership. Moreover, in societies in which reconciliation of work and family during midlife is less burdensome, labor market outcomes of women following different family trajectories converge. Our findings show that women’s fertility and partnership behavior are inevitably interrelated and jointly influence employment and earning patterns until later in life. The results imply that promoting equal employment opportunities could have long-lasting effects on women’s economic independence.

**Electronic supplementary material:**

The online version of this article (10.1007/s13524-020-00874-8) contains supplementary material, which is available to authorized users.

## Introduction

The increase of female employment was the most significant change in labor markets during the past century (Esping-Andersen 2009; Goldin 2006). However, women’s labor market attachment and earnings remain closely related to their family role. Mothers’ employment rates and wages lag those of men and childless women, even when work experience is controlled for. This “motherhood (earnings) penalty” is a well-established finding in many Western countries (e.g., Correll et al. 2007; Harkness and Waldfogel 2003; Sigle-Rushton and Waldfogel 2007a). Country-comparative research suggests that the strength of the motherhood effect on women’s employment and personal earnings is shaped by contextual factors, such as women’s opportunities to reconcile work and family (Abendroth et al. 2014; Budig et al. 2012, 2016; Cukrowska-Torzewska 2017; Gangl 2004; Gornick and Meyers 2003; Halldén et al. 2016; Harkness and Waldfogel 2003).

Building on the motherhood earnings penalty literature, we argue that diversifying family patterns in the second half of the twenty-first century (Elzinga and Liefbroer 2007; Kiernan 2004; Sobotka and Toulemon 2008) calls for a more refined analysis of the consequences of women’s family life courses for their labor market outcomes. Because of increasing union instability and the postponement of parenthood, the traditional family trajectory of early and lifelong marriage and rapid and repeated childbearing is replaced by a variety of emerging family trajectories. A simple distinction between mothers and childless women therefore no longer reflects reality.

Furthermore, the literature on motherhood and labor market outcomes tends to focus on women’s prime years of childrearing (i.e., ages 25–45). However, young mothers’ decisions to quit their job or reduce their working hours not only lower their current income but also compromise their future job prospects and wages (Davies and Joshi 1994; Killewald and Zhuo 2015; Sigle-Rushton and Waldfogel 2007b). In aging societies, later-life labor market activity is of growing importance. In particular, it is important to examine women’s *personal* earnings because these reflect economic independence and therefore reduced vulnerability, especially at later ages. Also, women’s personal economic activity indicates the (under)use of productive potential.

The purpose of the present study is to advance our understanding of the association between women’s family trajectories and later-life labor market outcomes. We answer two research questions. First, how are different family trajectories associated with women’s later-life employment on the one hand and personal earnings on the other? Second, to what extent does the association between women’s family trajectories and later-life labor market outcomes vary across countries?

This study makes three contributions to the literature. First, we apply a life course perspective to women’s family trajectory by capturing the occurrence, order, and timing of family events as a coherent chain of events rather than as separate incidents (Elder et al. 2004). The literature has mainly focused on specific elements of the family life course, such as the age at first birth or the occurrence of a divorce (Abendroth et al. 2014; Budig and England 2001; Gough and Noonan 2013; Miller 2011; Pienta 1999; however, a recent exception is Jalovaara and Fasang 2019). We show that the consequences of the fertility and partnership trajectories for women’s later-life labor market outcomes can be more fully understood by acknowledging their interplay and thus studying the family life course holistically.

Second, we contribute to the literature by assessing long-term consequences of family life decisions. Labor market outcomes after age 50 are strongly influenced by cumulative experience over the life course (Dannefer 2003; DiPrete and Eirich 2006; Mincer and Polachek 1974). The family life course may have lifelong imprint effects on employment and earnings. Alternatively, aging might level out the inequalities between women following different family life courses. For instance, after children leave the parental home, the care-burden difference between mothers and childless women diminishes, possibly leading mothers to reenter the labor market and close part of the earnings gap.

Third, we examine the link between family trajectories and women’s later-life labor market outcomes from a comparative perspective. We investigate whether the level of female labor force participation during women’s family formation years moderated the association between their family trajectory and later-life labor market outcomes. Our data cover 22 countries, representing all European regions. This is the first study into this topic with such a broad range of countries. We combine microlevel data from three major longitudinal surveys: the Generations and Gender Programme, the British Household Panel Survey, and SHARELIFE. All three surveys contain extensive retrospective information on fertility and partnership trajectories and on current employment and personal earnings. Our sample consists of women aged 50 to 59 in the early 2000s, who were born between 1943 and 1963.

## Women’s Family Trajectories and Later-Life Labor Market Outcomes

During the “golden age of marriage” in the 1950s and 1960s, a woman’s life course was predictable: she would marry during her early 20s, subsequently quit the labor market, and have multiple children. For instance, in Western Europe, the marriage bar—that is, strong or even institutionalized social norms—demanded that married women resign from work (Festy 1980; Hakim 2004). From the 1960s onward, postponement of childbearing, union disruption, and single living became more prevalent (Billari and Liefbroer 2010; Cherlin 2010; Hantrais and Letablier 1996; Sobotka 2004). Simultaneously, women’s labor market participation expanded rapidly. In this section, we discuss how the new variation in women’s family trajectories associates with the emerged inequality in women’s economic activity. We start by reasoning that a woman’s paid labor activities during midlife may predict her later-life employment and earnings. Next, we explain how her family trajectory may relate to the midlife labor market investments she is able or willing to make.

### Consequences for Later-Life Economic Independence

Investment of time and energy in paid work during midlife may relate to later-life labor market outcomes due to human capital accumulation and path dependency. First, earnings and employment opportunities accumulate throughout life according to acquired human capital in the form of work experience (Mincer and Polachek 1974; Polanchek 1995). Reduction of working hours or nonemployment during midlife therefore likely lowers wages in later life. If women leave the labor market, their skills might become outdated, or at least perceived as such by employers, which decreases women’s opportunity to find a well-paying, interesting, or challenging job later on (Correll et al. 2007).

Second, women’s past decisions and activities regarding work and family limit their future options and preferences by means of established rules, habits, and selective information (Abendroth et al. 2014; Heinz et al. 2009; Moen et al. 1994; O’Rand and Henretta 1982). For instance, women who devote their midlife to care work and therefore prefer nonemployment, part-time, or flexible employment (over higher pay) presumably continue that habit in later life, even when their children have left the parental home. Also, if these women have ambitions to revive, boost, or change their career, they face increased transaction costs. For instance, it takes time, effort, and confidence to acquire new skills and socialize into an unfamiliar work setting.

### Four Reasons Why Partnered Motherhood Could Stall Career Investment

**Fig. 1:**
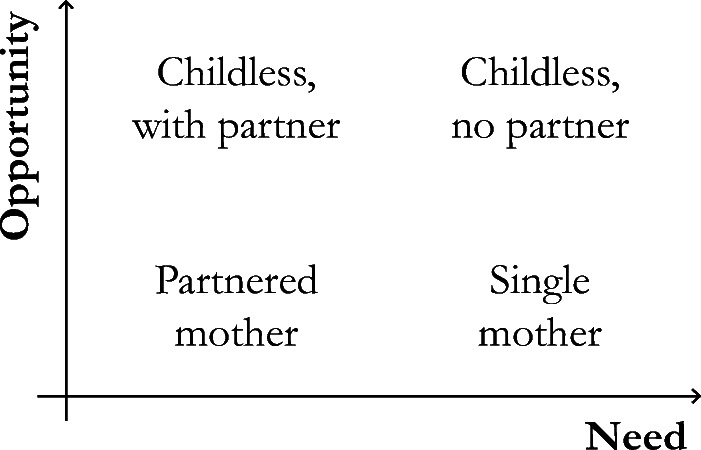
Family trajectories and women’s need and opportunity for labor market investments during their family formation years

If labor market investment during midlife is strongly related to later-life economic outcomes, the key question is, Which family trajectories increase chances of having a paid job? Here, we start by arguing that women with the most traditional family trajectory (early childbearing and long-term partnership) may be least likely to invest in employment during their childrearing years. In the subsequent subsection, we reason why delayed motherhood and childlessness increase women’s *opportunity* to earn money, whereas the absence of a partner increases women’s *need* to do so. These two mechanisms are visualized in Fig. 1.

In the cohort of women who have now entered later life (i.e., the cohort in our study), we expect that women who spent their entire midlife as mother and partner tended to focus on household and childcare activities, while their partners specialized in workplace activities. Normative expectations, discrimination, financial deliberation, and self-selection could have pulled these couples toward this gendered division of responsibilities.

First, whereas the societal role norm of “mother” predominantly regards caring, the role of “father” involves providing (Bielby and Bielby 1989; Blossfeld and Drobnič 2001; Myrdal and Klein 1956). Therefore, the male partner could easily establish identification with both employment *and* parenting roles, while the female partner experiences a tradeoff with these roles. Such role expectations were certainly dominant during the family-formation years of the women in this study and may still be relevant today.

Provoked by normative expectations, discrimination may be at play. The *job market signaling model* (Spence 1973) considers the hiring process as an investment under uncertainty in which the employer applies normative preconceptions to judge the productivity of a potential employee. Women who have children may be attributed with particular attitudes or characteristics related to their abilities and ambitions that imply lower productivity. Partnered mothers with family-related employment discontinuities in particular may signal less productivity to employers (Albrecht et al. 1999), which reduces the chance of being hired or being in high-quality employment with appropriate earnings. For instance, Correll et al. (2007) showed that mothers were considered less competent and passionate in their profession than childless women.

Third, economic motivations may strengthen the preference of couples with children for specialization. Assuming that partners pursue utility maximization, the most efficient strategy would be that the partner with the highest earnings capacity focuses on market production, while the other partner focuses on household work (Becker 1981). The former is most likely the man, as a result of differences in men’s and women’s initial investments in education and career, and because of gendered earning differences between and within sectors. Although this specialization strategy was common in marriage during the family formation phase of the women in this study, recent findings have suggested that specialization within couples today occurs mostly after childbearing (Juhn and Mccue 2017; Killewald and García-Manglano 2016; Killewald and Gough 2013; Langner 2015).

Finally, lower labor market investment of partnered mothers may be due to self-selection. For example, women might react to poor employment outcomes by choosing to invest their time in partnership and motherhood (Sigle-Rushton and Waldfogel 2007b) and have children at younger ages. Furthermore, Hakim (2000, 2003) suggested that women self-select based on preferences: for instance, women with a strong family aspiration may prefer limited or flexible employment over higher pay.

Combining these arguments, we expect that women whose family trajectory is dominated by motherhood and partnership have both the lowest need and the lowest opportunity during midlife to invest in paid labor. Hence, we expect their employment and earnings in later life to be lowest of all women. This results in our first hypothesis:*Hypothesis 1:* Women’s family trajectories characterized by having young children in combination with a partnership—that is, trajectories of partnered women who have children early, many children, and/or large spacing between children—are associated with lowest employment and lowest earnings in later life.

### Deviations From the Traditional Family Trajectory

In general, we expect that a family trajectory without a partner urges women to provide for themselves and possible children. In other words, absence of a partner increases women’s *need* to earn money. In addition, we expect that a family trajectory without children increases women’s *opportunity* to participate in the labor market because they are less bound in their decisions to role expectations or work division strategies. In this subsection, we discuss delayed motherhood, childless women with a partner, single mothers, and childless women without a partner (single-living women).

Partnered women’s later-life labor market outcomes may differ by timing of childbearing. The earlier women enter parenthood, the earlier they need to compromise on the accumulation of human capital. The longer women experience time constraints to invest in paid labor because of care work for small children, the longer they may need to stall career investments and the lower their opportunities to catch up career-wise later in their lives. By contrast, women who delay motherhood may have not only developed greater personal attachment to the labor market but also accumulated greater human capital prior to parenthood (Gough 2017). This may increase their opportunities to keep attached to the labor market during childrearing years or to reenter the labor market in high-quality and well-paid employment after family-related employment disruption. Based on these considerations, we formulate the second hypothesis:*Hypothesis 2:* Women’s family trajectories characterized by partnership and delayed childbearing are associated with higher employment and higher earnings later in life, compared with the aforementioned type of family trajectory (i.e., a partnered trajectory with early childbearing, many children, and/or large spacing between children).

Next, women without children have no care burden during midlife, which increases their *opportunity* to invest time and energy in labor market production. Therefore, we would expect that these women have higher later-life employment and earnings than mothers. Although partnered women without children have greater opportunities to invest in their career, they may still rely to some extent on the main providing role of their partner because of gendered norms regarding marriage or in anticipation of having a child. We therefore formulate the following hypothesis on how the expected gradient continues:*Hypothesis 3:* Women’s family trajectories characterized by partnership and no childbearing are associated with higher employment and higher earnings later in life, compared with the aforementioned type of family trajectories (i.e., trajectories characterized by partnered motherhood).

Furthermore, the absence of a partner, either as a result of (repeated) union dissolution or lifelong singlehood, increases women’s *need* to provide for themselves and their possible children. We expect a distinction between single women with children and without children.

Along with the decreased union stability, the number of single mothers increased (Cherlin 2010; Fokkema and Liefbroer 2008; Teachman et al. 2000). Compared with all other family trajectories, women who spend a considerable time as a single mother may experience the greatest *need* to provide. We expect that single mothers will be the least driven by normative expectations toward motherhood: because they simply need to care and provide simultaneously, they cannot afford to lower their time investment in labor market activity (Roman 2017). However, single mothers could have great difficulties reconciling work and family because they carry the childcare burden on their own. They might be forced by their situation to take on low-quality, precarious, low-paid, and/or low-intensity jobs (Christopher 2005; Nieuwenhuis and Maldonado 2018), which offer less opportunity for career development. Moreover, single mothers who reenter the labor market after a divorce might have lowered human capital accumulation due to a period of care leave when they had a partner and therefore have lower career prospects (Nieuwenhuis and Maldonado 2018). In sum, although single mothers might have a high need to provide, they have low opportunity.

We expect that a woman’s need to provide will have a stronger effect on later-life labor market outcomes than her opportunity. Although a need might be unavoidable, an opportunity permits freedom to choose. Therefore, we expect that single mothers (i.e., high need, low opportunity) will have higher later-life earnings than childless women with a partner (i.e., high opportunity, low need). We formulate the fourth hypothesis:*Hypothesis 4:* Women’s family trajectories characterized by childbearing and the absence of a partnership (single mothers) are associated with higher employment and higher earnings later in life, compared with the aforementioned type of family trajectories (i.e., trajectories characterized by partnered motherhood and trajectories characterized by partnership without children).

Finally, unpartnered women without children may perceive the greatest opportunity to invest in their professional career as their employment decisions cannot interfere with career ambitions of their partner (Verbakel et al. 2008) nor with care obligations toward children. Also, they have a clear need to provide for themselves. We would thus expect these women to have the highest employment rates and earnings later in life:*Hypothesis 5:* Women’s family trajectories characterized by the absence of a partnership and by childlessness (single living) are associated with highest employment and highest earnings later in life.

To summarize Hypotheses 1–5, we expect a *gradient* in later-life employment and earnings, ranging from the lowest expected outcomes for women following a traditional family trajectory of long-term partnership and early childbearing to the highest expected outcomes for unpartnered women without children.

## Context Variation in the Relationship Between Women’s Family Trajectories and Later-Life Labor Outcomes

In the previous section, we argue that women’s later-life employment and earnings depend on their opportunity and need for labor market investments during their family formation phase. Here, we reason that this family trajectory gradient in later-life employment outcomes depends on contextual circumstances (Elder et al. 2004; Heinz et al. 2009). More specifically, we argue that the gradient is weaker in countries that offered comprehensive support for maternal employment and hence where it was common for women to remain attached to the labor market during their family formation years.

In the 1970s and 1980s, when most women in our sample started their family life course, female employment levels differed strongly across Europe. Normative beliefs and policy regimes in Sweden, Denmark, and countries in eastern Europe showed the strongest support for maternal employment. By the mid-1980s, mothers in these countries were able to remain in full-time paid work with minimal career disruptions (Gornick et al. 1997; Pascall and Manning 2000). Indeed, female labor force participation was highest in the Scandinavian countries (e.g., 75% in Sweden) and in the satellite states of the Soviet Union (e.g., 78% in Estonia). However, support for maternal employment in other European countries, such as West Germany, the Netherlands, and the United Kingdom was more limited or discontinuous (Gauthier 1996; Gornick et al. 1997). In these countries, the childcare burden remained predominantly on parents, resulting in more traditional gender role patterns. Female labor force participation was moderate in western Europe and the United Kingdom, where between 43% (Belgium) and 55% (France) of women participated in the labor market. It was lowest in the southern European countries (e.g., 33% in Spain), where less than half of all women were in paid labor (all percentages from Generations and Gender Programme 2016).

Women’s opportunity to remain attached to the labor market during their family formation phase depends on contextual circumstances that likely enhanced female employment, such as progressive gender role attitudes and formalization of the care sector. First, women’s labor market participation depends on cultural norms about gender roles and gender equality as well as actual gendered practices within households (Fortin 2005). More gender-egalitarian norms may translate into a less gendered division of paid and unpaid work, with men doing a greater share of housework and care work and thereby increasing their female partner’s opportunities to invest in her career.

Second, societies differ in the extent to which care for children is *defamilialized*: that is, regarded as a public responsibility and provided by formal (i.e., paid or taxed) care services (Bettio and Plantenga 2004; Esping-Andersen 1999; Saraceno and Keck 2010; Tijdens 1993). The availability of such formal services is generally viewed as a precondition for women’s capacity to participate in the labor market (i.e., commodify themselves) and have a continuous career regardless of their family choices (Akgunduz and Plantenga 2018; Orloff 1993). In addition, the affordability of care services is critical. Especially for single mothers, the price of childcare could be too high compared with their single-income resource level (Moilanen et al. 2016; Saraceno 2011).

The provision of care by public services outside the home facilitates maternal employment by saving mothers’ time and energy (Mandel and Semyonov 2006; Misra et al. 2007). Also, formalization of the care system implies an expanded care sector, with increased numbers of jobs available in (female-dominated) care work, fostering women’s employment opportunities in later life (Prince Cooke 2011). In addition, the aforementioned cultural gender norms could amplify or even change the effects of family policies on women’s employment and earnings (Budig et al. 2012). Mothers benefit most in terms of earnings from parental leave and childcare policies in countries where attitudes support maternal employment.

Although public expenditures on childcare could lead to a lower midlife motherhood occupational status penalty (Abendroth et al. 2014), women in countries with extensive family provisions tend paradoxically to work more in female-typed occupations and hold fewer managerial positions (Mandel and Semyonov 2006). Moreover, not all family policies enhance women’s labor market opportunities (Misra et al. 2007). Most strikingly, extensive care leave provisions, called *supported familialism* by Saraceno and Keck (2011), could demotivate women to reenter the labor market after childbirth. Also, extensive care leave provisions may cause discrimination by employers against young women (Mandel and Semyonov 2006) because they expect women to have lower levels of productivity, on average. Employers could therefore be reluctant to hire young women and/or invest in women’s on-the-job training.

The previous discussion makes clear that women face considerable challenges in the labor market even in societies with extensive family policies. Still, we generally expect that in countries that facilitated the reconciliation of work and family activities, women have had greater opportunities to invest in their working careers independently from their family life course, leading to a weaker family trajectory gradient in later-life employment outcomes. We thus formulate our final hypothesis:

- *Hypothesis 6:* Family trajectory–related differences in women’s later-life employment and earnings are expected to be smaller in countries with higher female labor force participation during women’s family formation phase.

## Methods

### Data and Sample

Our study uses data from the first wave of the Generations and Gender Programme (Fokkema et al. 2016; Vikat et al. 2007), the fifteenth wave of the British Household Panel Survey (Perelli-Harris et al. 2015; University of Essex Institute for Social and Economic Research 2010), and the third wave of SHARE (SHARELIFE) (Börsch-Supan 2010; Schröder 2011). These surveys were collected between 2004 and 2013 and contain comprehensive fertility and partnership histories as well as information about current employment and earnings.

We restrict our sample to women aged 50 to 59 at the time of interview. Our analytical sample consists of 18,656 women from 22 European countries (Austria, Belgium, Bulgaria, Czech Republic, Denmark, Estonia, France, Georgia, East Germany, West Germany, Greece, Ireland, Italy, Lithuania, the Netherlands, Norway, Poland, Romania, Spain, Sweden, Switzerland, and the United Kingdom). Table 1 provides information about the year of data collection and sample size per country.

**Table 1:** Data sources and sample sizes per country

Country	Survey (year of data collection)	*n* (data source)	*n* (country total)
Austria	SHARELIFE (2008–2009)	91	91
Belgium	Generations and Gender Survey (GGS) (2008–2010)	675	1,077
	SHARELIFE (2008–2009)	402	
Bulgaria	GGS (2004–2005)	878	878
Czech Republic	GGS (2005)	908	1,208
	SHARELIFE (2008–2009)	300	
Denmark	SHARELIFE (2008–2009)	375	375
Estonia	GGS (2004–2005)	898	898
France	GGS (2005)	1,063	1,407
	SHARELIFE (2008–2009)	344	
Georgia	GGS (2006)	936	936
East Germany	GGS (2005)	187	251
	SHARELIFE (2008–2009)	64	
West Germany	GGS (2005)	675	886
	SHARELIFE (2008–2009)	211	
Greece	SHARELIFE (2008–2009)	525	525
Ireland	SHARELIFE (2007)	145	145
Italy	SHARELIFE (2008–2009)	299	299
Lithuania	GGS (2006)	746	746
Netherlands	GGS (2002–2004)	871	1,200
	SHARELIFE (2008–2009)	329	
Norway	GGS (2007–2008)	1,354	1,354
Poland	GGS (2010–2011)	2,403	2,782
	SHARELIFE (2008–2009)	379	
Romania	GGS (2005)	1,233	1,233
Spain	SHARELIFE (2008–2009)	253	253
Sweden	GGS (2012–2013)	838	1,028
	SHARELIFE (2008–2009)	190	
Switzerland	SHARELIFE (2008–2009)	206	206
United Kingdom	British Household Panel Survey (BHPS) Wave 15 (2005–2006)	878	878
Total	GGS	13,665	18,656
	SHARELIFE	4,113	
	BHPS	878	

### Dependent Variables

The first dependent variable is *employed* (vs. *not employed*) at the time of interview, measured by women’s reply to the question of what their current main activity is. Women answering “in employment or self-employment” were considered employed. Women in the category “not employed” comprised a diversity of activities, such as being unemployed, looking after the home or family, or being retired.

The second dependent variable is *personal net earnings*, measured as the natural log of annual earnings from a job or self-employment. Women were asked whether they received earnings from a job or business during the last 12 months, how often they received payment, and what the average net amount of payment was (i.e., the take-home pay). By multiplying the payment amount the appropriate times (adjusted for seasonal or otherwise not year-round work), we estimate the annual net earnings. Earnings data are missing in approximately 20% of our sample. We impute the earnings variable using multiple imputation (details can be found in the online appendix and Muller 2016). The results we present are not sensitive to the imputation of missing data on the dependent variable. Running the models presented on a reduced sample with nonimputed earnings (*n* = 9,500) yields identical results (available upon request).

We transform the earnings measure from national currencies to international comparable euros in three steps. First, we convert to the year 2008 (i.e., because most data were collected in 2008) by using the consumer price index to correct for inflation (World Bank n.d.-a). Next, we convert these national 2008 currencies to international comparable dollars using the purchasing power parity (PPP) conversion factor (World Bank n.d.-b). Last, we convert to PPP euros using the annual average exchange rate in 2008 between dollars and euros (i.e., 1 euro = 1.4708 U.S. dollars; Eurostat n.d.). Using PPPs allows us to make a more meaningful comparison between countries because it adjusts for differences in the cost of living (World Bank 2013). Logging ensures that the earnings distribution meets the assumption of normality and outlier effects are minimized. Multiplying the coefficients by 100 × (*e*^*b*^ – 1) gives the percentage change in earnings, given a one-unit increase in the independent variable.

### Independent Variables

At the micro level, the variable of interest is women’s family trajectory, which is measured as a sequence of yearly states from ages 18 to 50—that is, 33 chronological states. We specify eight possible states based on a combination of the age of the youngest child and partnership status: (1) no child, no partner; (2) no child, with partner; (3) youngest child aged 0–3, no partner; (4) youngest child aged 0–3, with partner; (5) youngest child aged 4–11, no partner; (6) youngest child aged 4–11, with partner; (7) youngest child aged 12+, no partner; and (8) youngest child aged 12+, with partner.

We consider the age of the youngest child because the care burden for that child is highest. We distinguish pre-primary, primary, and post-primary school age. We include biological and adoptive children, but we exclude stepchildren. Our assumption is that children for which mothers have prime responsibility have most impact on their employment need and opportunity.

The states “with partner” cover cohabiting and married partnerships with a male or female partner. We include unmarried coresident couples because cohabitation is viewed increasingly as an alternative or prelude to marriage (Hiekel et al. 2014). Also, we regard the situation of having children in a cohabiting union closer to having children within marriage instead of having children without a coresident partner.

At the macro level, we would ideally want to include indicators that explain country variation in women’s employment opportunities during their family formation phase (the 1970s and 1980s for women in our data). Important variables could be measures of gender role norms, the availability and affordability of formal childcare, the size of the public sector, and flexible labor market opportunities. Unfortunately, such detailed country-level data are not available, or are barely available, for the relevant period (the 1970s and 1980s), especially for the eastern European countries. Therefore, we focus on one general indicator that is available and reflects which countries were, at the time, forerunners in women’s (especially mother’s) employment and offered relatively comprehensive support for reconciliation of paid and unpaid work: the female labor force participation rate. The female labor force participation rate indicates the percentage of the female population aged 15–64 that is active on the labor market. We use 1980 data from the GGP Contextual Database (Generations and Gender Programme 2016), which is the first time point available. No macro data were available for East Germany.

### Control Variables

First, *educational attainment* positively affects employment, working hours, hourly wages, and ultimately earnings (Mincer 1975). Moreover, low education is associated with experiencing potentially disadvantageous events in the family trajectory, such as early parenthood and divorce (Härkönen and Dronkers 2006; Raymo et al. 2015). We use the International Standard Classification of Education (ISCED) to distinguish three levels of educational attainment: no or primary education (ISCED 0, 1, or 2), lower and upper secondary education (ISCED 3 or 4), and all types of tertiary education (ISCED 5 or higher).

Second, *age* relates to labor market outcomes resulting from human capital accumulation and selection effects (Murphy and Welch 1990). Also, age might relate to the prevalence of certain family trajectories because of cohort effects. We measure age in years at the time of interview.

Third, to disentangle retrospective and current family effects on labor market outcomes, we include two variables. One indicates whether the respondent had a *coresident child younger than 18 years* at the time of interview, and the other indicates whether she had a *coresident partner* at the time of interview.

Finally, given that earnings depend on the combination of the number of working hours and hourly wages, we include a dummy variable indicating whether the woman is employed *30 hours or more per week*. We cannot use a more detailed measure because the SHARELIFE data contain only this crude measure.

Descriptive statistics for all variables can be found in Table 2.

**Table 2:** Descriptive information per variable per country

			Educational Level					Family Trajectory Cluster								
Variable	Age (range = 50–59 years)	Birth Year (range = 1943–1963)	1 = Low	2 = Middle	3 = High	Coresident Children (1 = 1+ children)	Coresident Partner (1 = yes)	Child With Partner, Stretched	Child With Partner, Early	Child With Partner, Delayed	Single Mother	No Child, With Partner	No Child, No Partner	Employed (1 = yes)	Works Full-Time^a^ (1 = yes)	Log(Earnings)^a^ (range = (0,∞])
Austria	55.5	1953	30.8	55.0	14.3	6.6	87.9	25.3	39.6	14.3	11.0	2.2	7.7	39.6	31.9	9.3
Belgium	54.7	1954	36.6	32.5	30.9	10.6	76.3	16.3	35.4	16.0	17.9	9.8	4.6	58.6	30.9	9.4
Bulgaria	54.6	1949	27.7	48.0	24.4	19.5	78.6	13.9	54.4	11.3	12.9	4.2	3.3	56.6	45.4	7.9
Czech Republic	54.8	1951	27.7	61.9	10.4	8.3	65.2	13.6	40.6	9.9	21.6	7.3	7.0	63.5	54.1	8.6
Denmark	54.9	1954	12.0	33.1	54.9	8.3	83.2	22.1	29.9	21.6	14.4	7.5	4.5	83.7	61.3	9.6
Estonia	54.3	1950	13.7	49.1	37.2	10.9	65.6	25.2	34.3	7.5	26.0	3.7	3.5	74.5	69.3	8.6
France	54.7	1951	39.5	37.2	23.3	11.6	66.9	20.1	32.5	13.4	21.7	5.2	7.1	65.0	43.4	9.3
Georgia	54.2	1952	10.2	62.0	27.9	37.2	69.2	19.1	45.0	11.9	13.6	1.5	9.0	49.4	27.1	6.9
East Germany	54.6	1951	18.7	62.6	18.7	8.0	74.1	19.9	43.0	10.0	14.3	4.8	8.0	72.1	48.6	9.2
West Germany	54.5	1951	34.7	50.1	15.2	13.7	74.3	16.0	27.3	19.8	17.2	12.6	7.1	70.2	35.9	9.3
Greece	55.2	1954	37.7	42.3	20.0	4.8	81.9	18.7	48.4	12.8	8.2	6.5	5.5	36.8	35.1	9.3
Ireland	54.9	1952	24.8	26.9	48.3	18.6	75.2	40.0	16.6	17.2	13.8	4.8	7.6	46.9	30.3	9.3
Italy	55.1	1954	55.9	35.8	8.4	10.7	88.0	27.8	44.2	11.4	6.0	5.7	5.0	43.1	33.8	9.3
Lithuania	54.2	1952	10.3	63.3	26.4	15.6	52.4	15.8	29.1	11.8	27.8	5.5	10.1	72.4	60.1	9.6
Netherlands	54.5	1950	48.0	24.8	27.3	9.2	72.3	15.2	33.3	17.0	16.5	9.8	8.3	58.1	22.1	9.2
Norway	54.4	1953	16.0	48.2	35.8	18.1	72.5	27.4	28.6	12.9	21.3	4.9	5.0	88.6	56.3	9.8
Poland	54.9	1955	18.5	67.5	14.0	16.7	67.3	26.3	38.5	7.4	17.7	4.1	6.1	42.9	34.9	8.7
Romania	54.4	1951	52.3	39.2	8.5	17.5	73.6	19.2	45.2	10.5	14.5	8.3	2.4	41.4	28.5	7.7
Spain	55.2	1953	67.6	21.0	11.5	9.1	85.0	28.5	41.1	10.3	4.0	8.3	7.9	45.5	39.5	9.3
Sweden	54.7	1957	11.0	48.2	40.9	16.5	75.0	21.8	22.4	23.7	21.8	5.0	5.4	92.5	71.5	9.8
Switzerland	55.2	1953	26.2	62.6	11.2	6.8	74.8	16.5	34.5	16.0	16.0	5.8	11.2	69.9	23.8	9.5
United Kingdom	54.4	1950	28.1	39.4	32.4	8.2	69.7	19.8	29.4	15.8	20.5	5.6	9.0	66.0	37.0	9.4
Total	54.6	1952	27.8	48.3	23.9	14.4	71.2	20.5	36.1	13.0	18.1	6.1	6.2	61.2	42.4	9.0

^a^Values represent the percentage of full-time workers and average earnings among employed women.

### Analytical Approach

Our analytical approach consists of two parts. First, we create a typology of family trajectories using sequence cluster analysis. Second, we use this typology in regression models to predict variance in women’s later-life employment and earnings.

The strength of sequence analysis is that it provides a holistic view of trajectories, which allows us to determine trajectory patterns, taking into account ordering (sequencing), timing of family events, and duration of states (Cornwell 2015; Studer and Ritschard 2014). In sequence analysis, similarities between trajectories are expressed as distances—that is, trajectories that strongly resemble each other have a short distance to each other, whereas trajectories that are very different have a large distance. First, we calculate a pairwise distance matrix by using optimal matching with a constant substitution matrix and indel cost of 1 (Studer and Ritschard 2014). Next, we perform a hierarchical cluster analysis on this distance matrix using Ward’s method, which implies that sequences with the smallest distance from each other are clustered. To determine the most appropriate number of clusters, we consider two cluster cutoff criteria—namely, the average silhouette widths (ASW) and point biserial correlation (PBC) (Studer 2013)—as well as the construct validity of the cluster solution, by comparing the fit of regressions of the different cluster-solutions on earnings—Akaike information criterion (AIC) and Bayesian information criterion (BIC) (Han et al. 2017; Warren et al. 2015). We use the *TraMineR* package in R to perform the sequence cluster analysis (Gabadinho et al. 2011).

In the second step of our analysis, we use the family trajectory typology that results from the sequence cluster analysis to predict women’s later-life likelihood of being employed and the earnings of employed women.

To answer our first research question on how later-life employment and earnings systematically vary by women’s midlife family trajectory, we estimate pooled logistic (employment) and linear (earnings) models with country fixed effects. We include the family trajectory typology as a categorical variable (i.e., a set of dummy variables) to these models. To test Hypotheses 1–5, we contrast different family trajectory clusters with each other (by simply switching the reference category of the set of dummy variables). In these models, we add a variance-covariance matrix (VCE) cluster correction that adjusts standard errors for intracountry correlation (Huber 1967; Rogers 1993; White 1980). Because we are not interested in absolute income differences between countries, we center the dependent earnings variable by country data set; that is, we subtract the country-specific mean log(earnings) from the log(earnings) variable.

To answer our second research question regarding the extent to which the association between women’s family trajectories and later-life labor market outcomes varies across countries, we include interaction effects between the family trajectory typology dummy variables and country dummy variables in the regression models. Using a chi-square test for employment and an analysis of variance (ANOVA) for earnings, respectively, we test the interaction of the two categorical variables: country and family trajectory typology. This is an appropriate method to assess country effects in a study such as ours with relatively few Level 2 cases, i.e. only 22 countries (Bryan and Jenkins 2016; Cameron and Miller 2015; Snijders and Bosker 2012).

Finally, to examine whether the family trajectory gradient in later-life employment and earnings is moderated by the midlife level of female labor force participation in a given country, we test interaction effects between the family trajectory clusters and the 1980 macro-level female labor force participation rate.

## Results

### Typology of Family Trajectories

The cluster analysis of the sequence distance matrix results in a six-cluster solution. Although the two cluster cutoff criteria—ASW and PBC—indicate that the four-cluster solution would be optimal, the regression fit indices—AIC and BIC—are superior for the six-cluster solution (Table 3). We choose the six-cluster solution because in the four-cluster solution, 69.6% of women were assigned to one cluster; comparatively, in the six-cluster solution, 36.1% of women were in the largest cluster. Moreover, compared with the four-cluster solution, the six-cluster solution grasps more relevant detail of the family trajectory.

**Table 3:** Fit indices of several cluster solutions of family trajectories: Average silhouette widths (ASW), point biserial correlation (PBC), Akaike information criterion (AIC), and Bayesian information criterion (BIC)

Number of Clusters	ASW	PBC	AIC	BIC
4	0.47	0.76	21,708.0	21,810.8
5	0.26	0.51	21,709.1	21,819.2
6	0.28	0.49	21,688.4	21,805.9
7	0.28	0.51	21,690.3	21,815.1
8	0.19	0.41	21,692.2	21,824.4
9	0.20	0.41	21,691.8	21,831.3

*Notes*: AIC and BIC are estimated in a linear regression model with centered log(earnings) as dependent variable; independent variables are age in years, educational level, country and family trajectory, and a VCE cluster correction (data set).

**Fig. 2:**
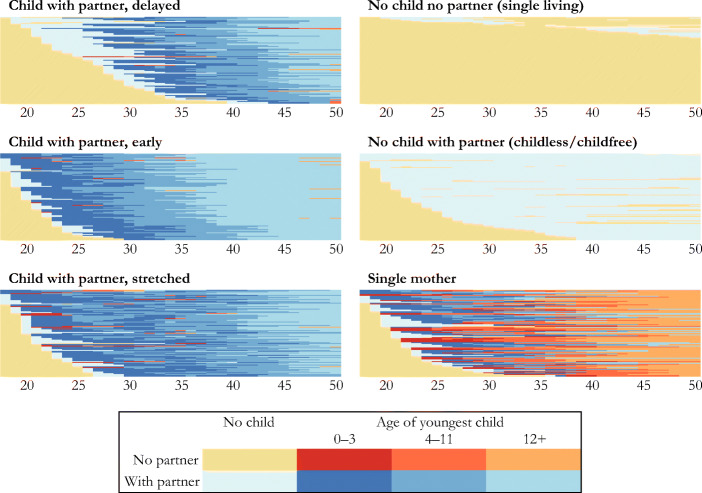
Sequence index plots of women’s family trajectories between ages 18 and 50 across 22 European countries. *n* = 18,656.

Figure 2 shows the sequence index plots of the six family trajectory clusters. We label each cluster based on its characteristics. We identify two types of traditional motherhood trajectories: *child with partner, stretched* (*CWP stretched*) and *child with partner, early* (*CWP early*). Women in these two clusters experienced the same sequence: they started living with a partner early in their adult life, had one or more children, and stayed together with their partner until at least age 50. The difference between the two clusters is the number and spacing of children. *CWP stretched* implies that women in this cluster had many children or a large time gap between births and therefore had an extended period of care burden.

The remaining four trajectories corresponded to the discussed deviations from the traditional, partnered motherhood trajectory. First, women in the *CWP delayed* cluster delayed partnering and motherhood. Second, we identify two clusters of childless women who spent most of their life (1) with a partner: *no child, with partner* (*NCWP*) or (2) without a partner: *no child, no partner* (*NCNP*)*.* A final cluster comprised women who experienced a substantial spell of *single motherhood*. Some of these single mothers started their trajectory traditionally; they coresided with a partner and had a child. However, after union dissolution, they continued living with their child(ren) without a partner. Other single mothers spent most of their life without a coresiding partner and raised their child(ren) on their own.

Most (69.6%) women in our sample were in one of the three *CWP* clusters. In addition, 12.3% of women were in one of the two childless trajectories (*NCWP* or *NCNP*), and 18.1% of women were in the *single mother* cluster. Table 2 shows the distribution of family trajectory clusters in total and by country.

### Estimating Employment and Earnings Differences Between Family Trajectories

Next, we include the derived family typology as a set of dummy variables in logistic regression models estimating whether women were in paid employment and in linear regression models estimating earnings among those women who were. Results on employment are presented in Table 4, and results on earnings are presented in Table 5.

**Table 4:** Logistic regression models predicting later-life employment of women

	1	2	3
Educational Level (ref. = middle education)			
Low education	–0.66***	–0.65***	–0.59***
	(0.06)	(0.06)	(0.12)
High education	0.86***	0.86***	0.99***
	(0.06)	(0.06)	(0.10)
Age in Years	–0.16***	–0.17***	–0.16***
	(0.02)	(0.02)	(0.02)
Current Characteristics			
Coresident partner (yes)		–0.12*	
		(0.06)	
Coresident child <18 (yes)		–0.14**	
		(0.05)	
Early and Midlife Family Trajectory Typology (ref. = child with partner, stretched)			
Child with partner, early	0.08	0.05	0.73*
	(0.05)	(0.05)	(0.30)
Child with partner, delayed	0.30***	0.30***	0.64
	(0.07)	(0.07)	(0.40)
No child with partner	0.11	0.07	1.70***
	(0.11)	(0.10)	(0.43)
Single mother	0.13	0.03	0.93*
	(0.09)	(0.09)	(0.44)
No child, no partner	–0.06	–0.18	1.30*
	(0.12)	(0.12)	(0.53)
Country-Level Variables and Interactions (ref. = female labor force participation (FLFP) 1980 × child with partner, stretched)			
FLFP 1980			2.37*
			(0.95)
FLFP 1980 × Child with partner, early			–1.22*
			(0.50)
FLFP 1980 × Child with partner, delayed			–0.40
			(0.67)
FLFP 1980 × No child, with partner			–2.59***
			(0.68)
FLFP 1980 × Single mother			–1.19^†^
			(0.70)
FLFP 1980 × No child, no partner			–2.26*
			(0.88)
Constant	9.54***	9.91***	7.43***
	(1.18)	(1.19)	(1.38)
*n*	18,656	18,656	18,405

*Notes*: Standard errors are shown in parentheses. The reference category for educational level is middle education. The reference category of family trajectory typology is child with partner, stretched. VCE cluster correction (data set) is included in all models. Country dummy variables are included in Model 1 and Model 2 (country coefficients are not shown in the table).

^†^*p* < .10; *p < .05; **p < .01; ***p < .001 (two-sided tests)

**Table 5:** Linear regression models predicting later-life earnings of employed women

	1	2	3
Educational Level (ref. = middle education)			
Low education	–0.29***	–0.24***	–0.28***
	(0.04)	(0.03)	(0.04)
High education	0.37***	0.34***	0.35***
	(0.03)	(0.04)	(0.03)
Age in Years	–0.01	–0.00	–0.01
	(0.00)	(0.00)	(0.00)
Current Characteristics			
Coresident partner (yes)		–0.03	
		(0.03)	
Coresident child <18 (yes)		–0.04	
		(0.03)	
Working hours 30+ (yes)		0.55***	
		(0.07)	
Early and Midlife Family Trajectory Typology (ref. = child with partner, stretched)			
Child with partner, early	0.06*	0.03^†^	0.03
	(0.02)	(0.02)	(0.10)
Child with partner, delayed	0.10**	0.08**	0.22
	(0.02)	(0.02)	(0.14)
No child, with partner	0.17***	0.10**	0.47*
	(0.04)	(0.03)	(0.22)
Single mother	0.14***	0.06*	0.22
	(0.02)	(0.02)	(0.18)
No child, no partner	0.22***	0.11**	0.73*
	(0.05)	(0.03)	(0.27)
Country-Level Variables and Interactions (ref. = female labor force participation (FLFP) 1980 × child with partner, stretched)			
FLFP 1980			–0.11
			(0.15)
FLFP 1980 × Child with partner, early			0.06
			(0.17)
FLFP 1980 × Child with partner, delayed			–0.19
			(0.22)
FLFP 1980 × No child, with partner			–0.48
			(0.36)
FLFP 1980 × Single mother			–0.13
			(0.28)
FLFP 1980 × No child, no partner			–0.82^†^
			(0.42)
Constant	0.21	–0.25	0.33
	(0.25)	(0.21)	(0.31)
*n*	11,415	11,255	11,234

*Notes:* Standard errors are shown in parentheses. Sample contains employed or self-employed women only. Dependent variable: centered log (earnings). The reference category of educational level is middle education. The reference category of family trajectory typology is child with partner, stretched. VCE cluster correction (data set) is included in all models. Country dummy variables are included in Model 1 and Model 2 (country coefficients are not shown in the table).

^†^*p* < .10; *p < .05; **p < .01; ***p < .001 (two-sided tests)

The first model in Table 4 shows that whether women aged 50–59 in Europe were in *paid employment* increases with level of educational attainment and decreases with age. To examine differences between family trajectories, we first perform a chi-square test assessing the joint effect of all family trajectory dummy variables, which is significant (χ^2^(5) = 41.61, *p* < .01). This implies that whether women are employed in later life differs significantly between family trajectory types.

Next, we examine whether the results regarding employment were in line with Hypotheses 1–5 by taking different family trajectories as reference category (detailed results available upon request). This is generally not the case. Women whose family trajectory was characterized by partnership and delayed childbearing (i.e., the *CWP delayed* cluster) had the highest employment rate. Differences between women in the other groups were relatively small and mostly nonsignificant.

Results in Model 2 of Table 4 show that the differences in employment between the family trajectory clusters remain largely the same when current family situation (i.e., whether women had a partner and/or a child in the household at the time of the interview) is controlled for.

**Fig. 3:**
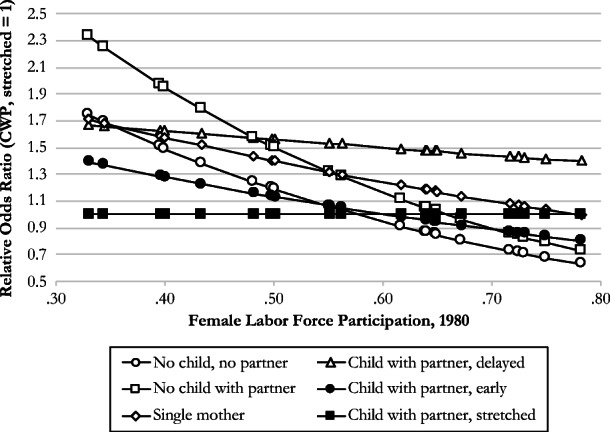
Relative odds ratio to be employed for women aged 50–59, by type of family trajectory and level of female labor force participation in 1980 (*child with partner, stretched* = 1). Coefficients are exponentiated (based on Table 4, Model 3). Traditional trajectories have a solid fill; deviations have no fill.

Last, we find significant variation in the effect of family trajectory between countries (χ^2^(11) = 10,857.60, *p* < .01). In Model 3, we add an interaction between the female labor force participation rate in 1980 and the family trajectory clusters. Several interaction terms as well as the combined interaction effects are statistically significant (χ^2^(5) = 23.51, *p* < .01). To facilitate interpretation of the interaction effect, we graph the predicted employment rate for all trajectory clusters across the levels of female labor force participation observed in the sample of countries (Fig. 3). Results are in line with the hypothesis. In countries with a low level of female labor force participation in 1980, differences in paid employment at ages 50–59 were relatively large between the family trajectory clusters, with women in the *CWP stretched* cluster having the lowest employment. In countries with a high level of female labor force participation in 1980, differences in paid employment at ages 50–59 between women with different family trajectories were smaller.

**Fig. 4:**
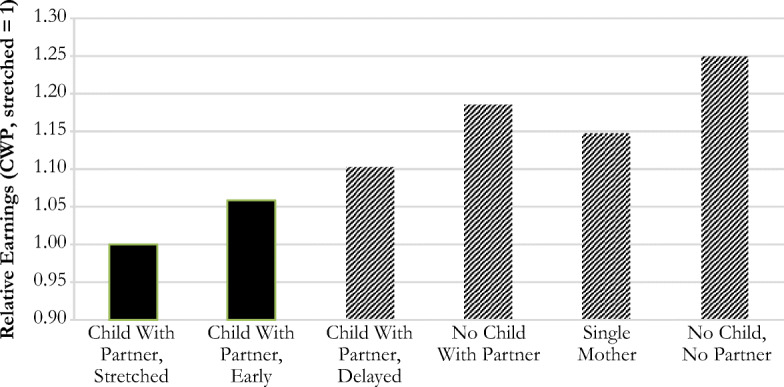
Relative earnings of women employed at age 50–59 by type of family trajectory (*child with partner, stretched* = 1). Coefficients are exponentiated (based on Table 5, Model 1). Traditional trajectories have a solid fill; deviations are striped.

Table 5 shows models predicting later-life *earnings* among women in paid employment. Earnings increased with educational attainment, but we observe no clear age pattern. To examine the overall effect of the family trajectory typology, we perform an ANOVA test assessing the joint effect of all family trajectory dummy variables, which is significant (*F*(5,26) = 9.33, *p* < .01). Subsequently, we test Hypotheses 1–5 by taking different family trajectories as reference category (again, by contrasting different trajectories against each other; detailed results available on request). Figure 4 presents the relative earnings by family trajectory cluster, based on Model 1 in Table 5. In general, we find that the order of the clusters corresponds with the expectations in Hypotheses 1–5. However, not all differences between clusters are statistically significant.

In line with the first hypothesis, our findings indicate that being a mother with a traditional family trajectory—that is, having children at a young age and a lifelong partner—penalizes women most in terms of personal earnings. Furthermore, women with the trajectory *CWP delayed* earned, on average, 10.2% more in later life than women with trajectory *CWP stretched*. This implies that in line with the second hypothesis, among partnered mothers, the longer the time women spent with dependent children, the lower their earnings in later life.

Next, we find that *NCWP* and *single mothers* indeed had higher earnings than all partnered motherhood trajectories (in line with Hypotheses 3 and 4). However, s*ingle mothers* and *NCWP* did not significantly differ from each other, contrary to our expectation (Hypothesis 4). Last, women with the *NCNP* trajectory had significantly higher earnings than all other women, as expected (Hypothesis 5), except for women with the *NCWP* trajectory, which was not expected. For instance, on average, women with the *NCNP* trajectory earned 24.9% more annually than women in the *CWP stretched* trajectory. Combining the insights from Hypotheses 1–5, we find a gradient in women’s later-life earnings according to their family trajectory as shown in Fig. 4.

Model 2 of Table 5 shows that differences in earnings between trajectory clusters become somewhat smaller only when controlled for current partner status, currently having a child in the household, and full-time working hours. This suggests that the differences between clusters at least partially result from differences in earning potential between women and not just from differences in the number of working hours.

**Fig. 5:**
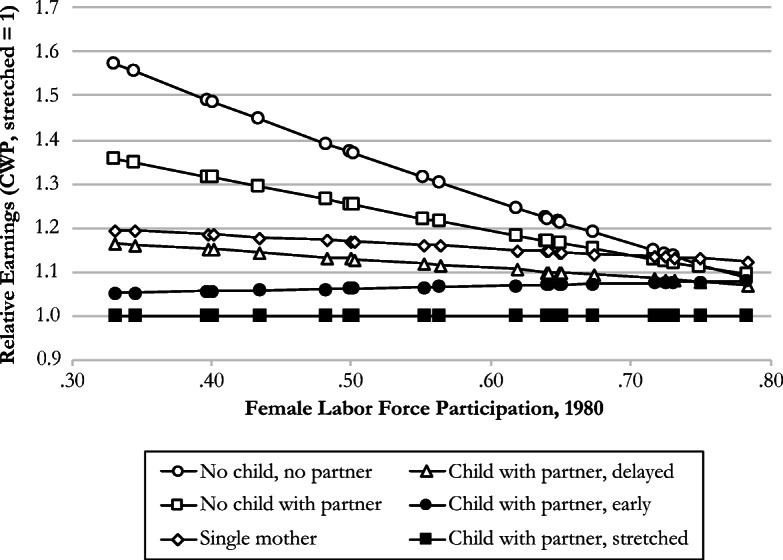
Relative earnings of women employed at age 50–59 by type of family trajectory and level of female labor force participation in 1980 (*child with partner, stretched* = 1). Coefficients exponentiated (based on Table 5, Model 3). Traditional trajectories have a solid fill; deviations have no fill.

Finally, we find significant variation in the effect of family trajectory on earnings between countries (*F*(43,75.7) = 15.82, *p* <.01). In Model 3 of Table 5, we examine those country differences by adding interaction effects between trajectory clusters and the country-level female labor force participation ratio in 1980. Hypothesis 6 states that higher female labor force participation in a given country at the time of women’s family formation (1980) would be associated with smaller differences in later-life earnings between women with different family trajectories. The overall picture, visualized in Fig. 5, is in line with this hypothesis. In countries with low levels of female labor force participation differences in earnings between women with different family trajectories are substantial, but they are small in countries with high levels of female labor force participation. However, only the difference between the *NCNP* cluster and the *CWP stretched* cluster statistically significantly weakens. In sum, our results are in line with Hypothesis 6, but only the difference between the two extreme clusters declines significantly by country’s female labor force participation rate.

## Discussion and Conclusion

In this study, we examine whether women’s family trajectories have long-term consequences for employment and personal earnings. Using data on 22 European countries, we derive six family trajectory types based on timing and number of children, and timing and number of partnerships between ages 18 and 50. Subsequently, we use this typology to predict differences in employment and earnings among women aged 50–59. Our work extends previous literature on the motherhood earnings penalty by examining the intertwined fertility and partnership trajectory, studying their long-term effects, and taking a comparative perspective.

Our first main finding is that contrary to what prior research on the motherhood earnings penalty suggests (e.g., Harkness and Waldfogel 2003; Sigle-Rushton and Waldfogel 2007a), there is no strict employment and earnings divide between mothers and nonmothers. Rather, we find little variation in women’s later-life employment, and we find evidence for a gradient in women’s earnings based on the diversity in their family trajectories. Especially the earnings of single mothers and partnered women who delayed motherhood are similar to those of childless women. Our results indicate that an earnings penalty exists mostly for women with the most traditional motherhood trajectory of lifelong coresident partnership, early motherhood, and multiple children. On the other hand, women who live without a partner and without children the majority of their life (i.e., single living) have the highest earnings at the end of their careers.

We find that the higher women’s *need* to provide during midlife (i.e., the more time without partner) and the more *opportunity* to invest in the labor market (i.e., the more time without dependent children), the higher their later-life earnings. However, we do not find evidence that the need to provide is more important than the opportunity: the earnings levels of single mothers (high need, low opportunity) and partnered childless women (low need, high opportunity) do not differ.

Although we observe large earnings differences between women according to their family trajectory, we find relatively small differences in employment. Partnered women who delayed childbearing had the highest employment rate, suggesting that their delayed pattern of childbearing increased their chances of remaining attached to the labor market. However, contrary to our expectations, we find that single mothers and childless women (with or without children) did not have higher employment rates than partnered mothers.

The second main finding of this study is that the midlife family trajectory has long-lasting consequences for women’s personal earnings and employment beyond the childrearing years. Our findings of a family trajectory gradient in women’s later-life earnings, even when we take into account their current family situation, strengthen our confidence that women’s family trajectories have long-lasting implications for their earnings. This result puts previous findings on midlife motherhood earnings penalties in a broader life course perspective (e.g., Gangl and Ziefle 2009). The accumulating nature of labor market earnings makes it vital for women to have the opportunity to reconcile work and family activities in order to be more economically independent later in life.

Our third main finding is that context matters. Confirming our hypothesis regarding country variation, women’s family trajectory gradient in employment and earnings was smaller in countries with higher female labor force participation in 1980, when women in our study had to reconcile care work and career investments. This suggests that in societies in which reconciliation of work and family during midlife is less burdensome, labor market outcomes of women following different family trajectories converge and hence decrease economic inequality between women until the end of their careers.

In addition to these contributions, our study leaves a number of interesting questions unanswered. First, an important topic for future research would be to disentangle the effects for working hours and hourly wages. We show that working full-time hours does not fully mediate the effect of family trajectory on later-life earnings, suggesting that earnings of women with different trajectories vary because of both differences in working hours and in wage per hour. Our data do not allow a more detailed analysis of this matter, nor can we decompose the comparative findings in effects of working hours and hourly wages.

Second, a main strength of our analytical approach is that it appreciates the full family trajectory, the interdependence of life events, and their combined meaning. At the same time, we cannot pinpoint whether specific aspects of these trajectories (e.g., occurrence, timing, and/or duration of family spells) matter. Future research could answer such questions, needing highly detailed panel family and earnings data and using a fixed-effects approach.

Moreover, such prospective panel data would be beneficial because these data do not suffer from any recall bias. For the current study, we use retrospective family trajectory data. Although people usually do not forget when their children were born, they might not report relationships, especially those that were brief. This study therefore potentially underestimates the number of partnerships ever entered or dissolved. Longitudinal, career-long data are available in only a handful of often-studied countries, however, making these data hard to apply in a comparative perspective.

Third, we focus only on women and their personal earnings. Retrospective partner information is lacking in our data sets, but it would be interesting to examine the role of partner characteristics.

Last, as we indicated earlier, ideally we would have used more detailed macro variables in our comparative analysis. This would allow us to disentangle the relevance of, for instance, different family policies, labor market characteristics, and societal role norms. However, more specific indicators (e.g., the availability and affordability of formal childcare, the size of the public sector, gender inequality, gender role norms, and flexible labor market opportunities) are barely available for the 1970s and 1980s. Most information is available from the 2000s onward, especially in the former Soviet countries. Our strongest recommendation for future research is, therefore, that we need better historical data on family policies and other relevant societal aspects to further unravel the mechanisms behind women’s divergent levels of later-life economic resilience.

## Electronic supplementary material

ESM 1 (PDF 60.6 kb)

## Data Availability

All data sets used are publicly available from GGP, SHARE, and BHPS. The GGP earnings data set prepared specifically for this study is available on the GGP website. A publication package including all syntaxes is available on request from the first author.
