# Supplementary material for: The Long-Term Costs of Family Trajectories: Women’s Later-Life Employment and Earnings Across Europe
Source: Demography. 2020 Apr 23;57(3):1007–34. doi: 10.1007/s13524-020-00874-8 (PMC7329756; doi:10.1007/s13524-020-00874-8)
Supplement: Supplementary file 1 — (PDF 60.6 kb) [file 13524_2020_874_MOESM1_ESM.pdf]

Online Appendix

Earnings variable: harmonization of datasets and handling of missing data

In this appendix, we present additional information on how the earnings variable was created and how missing earnings data were handled. To retain as much information as possible, missing earnings values were imputed using multiple imputation. Percentage missings ranged from 2.49% in France (GGP) to 24.83% in Czech Republic (GGP). In the multiple imputation model a large selection of control variables was used covering personal information (e.g., gender, age), educational level, family characteristics (e.g., coresident partner and coresident young children), house characteristics, health information, possessions, job characteristics and financial indicators (e.g., "difficulty to meet ends").

In all countries, earnings information was collected with survey questions, except in Sweden (GGP) and Norway where this information was derived from register data. The GGP questionnaires asked whether the respondent received earnings from a job or business during the last 12 months, how often the respondent received payment, and what the average net amount of payment was (i.e., the take-home pay). Respondents were asked how often they received payment, to adjust for seasonal or otherwise not-year-round work, however most often this was 12 times (i.e., monthly). We assume that respondents included tips, overtime pay and other employer cash benefits in their reported earnings. By multiplying the monthly earnings the appropriate times, we estimated the annual net earnings. If respondents were not able or willing to indicate an exact amount, they were asked in which range their earnings lay. We replaced such indicated bands by the median of the earnings of respondents who did indicate an exact value within that band. We used the median rather than the mean, because earnings are not normally distributed within bands but positively skewed.

The SHARELIFE questionnaire only asked about monthly earnings and not about the number of times that amount was paid over the last 12 months. Therefore, we had to assume that this was year-round work. As a robustness check, we compared the earnings distributions in the GGP and SHARELIFE samples of six countries for which both GGP and SHARELIFE data were available (Belgium, Czech Republic, France, Netherlands, Poland and Sweden). We did not find large differences.

Four GGP countries (Poland, Estonia, Sweden and Norway) did not provide monthly earnings information, but rather the net total annual income received. This measure also includes other income sources than earnings from a job or self-employment, such as social benefits. In order to minimize measurement difference between the datasets, we checked whether respondents were employed with additional survey questions. These questions regard current activity status (employed or not), number of working hours per week and income payment types received. If respondents were not employed, we assumed their earnings to be zero.

We assume that the lion's share of the total income of women with earnings from a job or self-employment depends on their earnings. Therefore, we expect that the ranking of the two income definitions is roughly the same. However, we do realize that especially at the bottom of the income distribution, the ordering between the net earnings and total net income could be different due to social benefits.
